# Clinical analysis in immunocompetent and immunocompromised patients with pulmonary cryptococcosis in western China

**DOI:** 10.1038/s41598-020-66094-7

**Published:** 2020-06-10

**Authors:** Junyan Qu, Xiaoli Zhang, Yang Lu, Xijiao Liu, Xiaoju Lv

**Affiliations:** 1 Center of Infectious Disease, West China Hospital, Sichuan University, 37 Guoxue Lane, Chengdu, 610041 China; 2 Pathology department, West China Hospital, Sichuan University, 37 Guoxue Lane, Chengdu, 610041 China; 3 Radiology Department, West China Hospital, Sichuan University, 37 Guoxue Lane, Chengdu, 610041 China

**Keywords:** Fungal infection, Radiography, Antimicrobial therapy

## Abstract

Cryptococcosis is a systemic infection and it may occur in immunocompromised and immunocompetent hosts. In order to better understand the clinical characteristics of patients with PC in different immune status, we retrospectively investigated the clinical, radiological, and treatment profiles of immunocompetent and immunocompromised patients with PC during a 10-year period (2008–2017). As a result, out of 136 patients, 94 (69.1%) were immunocompromised hosts. For the PC patients without CNS involvement, higher percentage of immunocompetent patients (39.5%, 15/38) had asymptomatic presentation than immunocompromised patients (6.3%, 3/48) (P < 0.05). Multiple pulmonary nodules (72.7%, 56/77), ground-glass attenuation/interstitial changes (94.4%, 17/18) and cavitation (88.6%, 31/35) were significantly frequent in immunocompromised patients (P < 0.05). A total of 47 patients were misdiagnosed as tuberculosis or tumors based on CT signs. PC was likely to be misdiagnosed as tuberculosis in immunocompromised patients (88.2%, 15/17), and tumor was more likely to be considered in immunocompetent patients (43.3%, 13/30). Immunocompetent patients accounted for 80% (24/30) of patients with definite diagnosis on surgical lung biopsy. Fluconazole monotherapy can achieve good clinical outcome in most PC patients without central nervous system (CNS) involvement (91.5%, 54/59). After 3 months of treatment, 92.7% (38/41) patients have improved imaging findings. In conclusion, PC has diverse imaging manifestations and it is easily misdiagnosed. Lobectomy should be carefully selected in immunocompetent patients with a single lung lesion. Fluconazole monotherapy is preferred for PC patients without CNS involvement.

## Introduction

Cryptococcosis is a fungal disease with worldwide distribution caused by members of the *Cryptococcus gattii/neoformans* species complexes. There were approximately 225,000 new cryptococcal meningitis cases occur globally each year with significant mortality, especially among acquired immune deficiency syndrome (AIDS) patients^1^. *Cryptococcus* is widely distributed in nature and can be found in bird droppings, soil and decaying wood^2^. The respiratory tract is the primary portal for cryptococcal invasion, pulmonary cryptococcosis (PC) may occur after inhalation of *Cryptococcus* spores^3^. Epidemiological study have shown that the incidence of PC has increased more than six times between 1999 and 2006 in British Columbia, Canada, which has the largest number of *Cryptococcus deuterogattii* infections reported worldwide^4,5^. In recent years, cryptococcosis has been on the rise in China because of better diagnostics and prior publication bias^6^. *Cryptococcus* may disseminate to central nervous system (CNS) and other organs via blood transfusions. Immune function plays a vital role in the development of cryptococcosis. Although immunocompromised hosts are more susceptible to PC, it can also occur in immunocompetent subjects^7^. Recent researches also found these apparently immunocompetent patients may have potential immune genetic defects^8–10^. PC is often misdiagnosed as bacterial pneumonia, tuberculosis or lung cancer because of the similar clinical manifestations and radiological characteristics. Previous reports on the characteristics of cryptococcosis in China have focused on the patients from southeast and north of China^11,12^. Data about PC from western China is sparse. Due to the different climatic conditions in the eastern and western China, the disease characteristics maybe different. With the development of new diagnostic techniques and the increase in the number of immunocompromised individuals in recent years, we need to better understand the clinical characteristics of patients with PC in different immune status.

In this study, we compared the demographic features, clinical presentations, radiographic findings, therapeutic strategies and therapeutic outcomes in immunocompetent and immunocompromised PC patients who were admitted to a university hospital from Jan 2008 to Dec 2017 in western China.

## Materials and Methods

### Patients

From Jan 2008 to Dec 2017, the patients with a diagnosis of PC at hospital discharge were retrospectively reviewed in West China Hospital, Sichuan University, China (a 4,300-bed academic tertiary hospital). Inclusion in the final study group required the diagnosis of PC as defined as follows: (1) clinical and radiographic findings consistent with PC; and (2) histological presence of the organism in lung specimens, and/or isolation of *Cryptococcus* from respiratory secretions and/or blood specimens without other suspected etiologies, and/or positive result of a serum cryptococcal capsular polysaccharide antigen (CrAg) test. Lumbar punctures were performed in the patients with positive blood *Cryptococcus* culture or positive CrAg test or patients with clinical manifestations of meningitis. The diagnosis of cryptococcal meningitis was made if cerebrospinal fluid (CSF) culture for *Cryptococcus* and/or CSF India ink stain were positive. Exclusion criteria were age under 14 years, pregnant mothers, suspected or confirmed co-infection with other pathogens. Follow-up data was obtained through telephone calls and outpatient department visits. The last follow up was on December 31, 2018. The following data of age, sex, underlying diseases, initial clinical presentations, duration of initial symptoms to diagnosis, laboratory data, radiographic findings, therapeutic strategies and therapeutic outcomes were extracted and verified from the medical records by two authors independently.

### Laboratory studies

Laboratory tests such as blood routine, blood biochemistry, T lymphocyte subset, serum immunoglobulin and human immunodeficiency virus (HIV) test were performed. CSF was sent for white blood cell count, glucose, chloride, total protein, India ink stain, CSF CrAg test, tuberculous (TB) smearing, TB DNA, fungal and bacterial cultures. Fungal culture was performed on Sabouraud dextrose agar (SDA). CrAg test was performed using Latex Cryptococcal Antigen Detection System (Immuno-Mycologics, Inc., Norman, USA). T lymphocyte subset was measured using flow cytometry. The levels of serum immunoglobulin (Ig) G, IgA and IgM were measured using immunoturbidimetry.

### Group classification

The patients were divided into two groups (immunocompetent patients and immunocompromised patients group) according to their immune status. The patients who are classified into the immunocompromised group must meet at least one of the following conditions:^7^ (1) Any two values of serum IgG, IgA or IgM were below the lower limit of normal detection values; (2) The percentage of CD4 cells was <30% or CD4+ T lymphocyte count was <350/μL or the ratio of CD4/CD8 < 1. (3) Peripheral absolute neutrophil count was <2.0 × 10^4^ cells/mL or absolute lymphocyte count was <1,000 cells/mL. (4) Patient has at least one of the following conditions: use of immunosuppressive drugs or corticosteroids, uncontrolled diabetes mellitus, hematological malignancies, solid tumor, HIV infection, history of organ transplantation, severe respiratory system limitation or connective tissue disorders, such as rheumatoid arthritis and systemic lupus erythematosus.

### Histology

Of the 136 cases, 72 cases were diagnosed by histopathology. A total of 30 cases received surgical biopsy, 31 cases underwent transthoracic needle biopsy and 11 cases underwent transbronchial lung biopsy (TBLB). Tissues were fixed in 10% neutral-buffered formalin for histological examination, embedded in paraffin and cut into 4 mm sections. Sections were stained with hematoxylin-eosin and examined by light microscopy. Histochemically stained with periodic acid-Schiff (PAS), mucus card Red (Mc) and Gomori Methenamine Silver (GMS) were performed in every sample and some tumor markers were performed in part of the samples according to initial pathological findings. Acid-fast staining and *M. tuberculosis* examination by fluorescence quantitative polymerase chain reaction (qPCR) (Zhishan biotechnology co., LTD, Xiamen, China) were also performed in part of the samples.

### Radiological assessment

All PC patients were scanned by 64-row multi-slice spiral computed tomography (CT) (SOMATOM definition AS+, Siemens) in our hospital. Consecutive 5-mm thick sections were obtained from the lung apices to the lung bases during inspiration in the supine position. High resolution CT scans were obtained with 1-mm collimation in some patients. All images were reviewed independently by two thoracic radiologists who were unaware of the patients’ immune statuses and clinical symptoms. Several radiological findings were recorded including nodule or mass, pulmonary cavity, patchy consolidation, ground glass opacity, mediastinal lymph node enlargement, pleural effusion, pleural incrassation or conglutination. The distribution of the lesion in the lungs was also collected. The brain CT or magnetic resonance imaging was performed if the patients had CNS infections. After a definite diagnosis in our hospital, some patients returned to the local hospital for further treatment and follow-up. We obtained follow-up CT examinations of some patients after treatment. Improvement of CT images was defined as a reduction in the number and size of lesions. Complete radiologic remission was defined as complete disappearance of the lesions.

### Treatment strategies

PC patients were treated with fluconazole (200–400 mg per day IV or orally) or Itraconazole (200 mg twice per day orally). Some patients initially diagnosed with lung cancer underwent surgical lobectomy. According to the guidelines for the management of cryptococcal disease and our experiences^13,14^, the patients with CNS involvement were treated with two antifungal agents (amphotericin B/amphotericin B liposome and 5-flucytosine) or three antifungal agents [amphotericin B (0.5–1 mg/kg per day IV)/amphotericin B liposome (3 mg/kg per day IV), 5-flucytosine (100 mg/kg per day orally) and fluconazole (800 mg per day IV)] for 8–12 weeks of induction therapy, and then fluconazole (400 mg per day IV or orally) for 8 weeks of consolidation therapy followed by fluconazole (200 mg per day orally) for more than 6 months of maintenance therapy. Fluconazole would be replaced by voriconazole (loading dose, 6 mg/kg twice per day; maintenance dose, 4 mg/kg twice per day IV or orally) if the initial treatments were not effective. In the subjects with HIV infection, the consolidation and maintenance periods would be prolonged.

### Clinical outcome evaluating criteria

Based on clinical manifestations, laboratory tests, imaging findings and mycologic evidence, clinical outcome was classified as follows: (1) Improvement was defined as a complete or partial response to therapy for 1 year after receiving medical treatment or surgery. (2) Failure was defined as death or disease progression or persistence for 1 year with medication. For postoperative and asymptomatic patients, the assessment of antifungal response was mainly based on imaging findings indicating continuous improvement and absence of new lesions.

### Statistical analysis

Statistical analyses were performed using SPSS version 22.0 for windows (SPSS Inc., Chicago, USA). Shapiro-Wilk normality test was conducted for all quantitative variables^15^. Significance testing was carried out using Student’s t test for normally distributed data and Chi-square test or Fisher exact test for categorical variables. A two-tailed P < 0.05 was considered statistically significant.

### Ethical approval and informed consent

This study was conducted in accordance with the amended Declaration of Helsinki and was approved by the Ethics Committee of West China Hospital, Sichuan University, which waived the informed consent requirement because all the data in this study were routinely obtained.

## Results

### Patient characteristics

From January 2008 to December 2017, a total of 136 PC patients (mean age 51.91 ± 15.10 years; 87 males) were included in this retrospective study. The demographic and clinical features of these patients were summarized in Table 1. High peak age of the PC patients was from 31 to 60 years old (92/136, 67.6%) regardless of the immune status. CNS involvement was more common in immunocompromised patients than in immunocompetent patients (P <0.05). For the patients without CNS involvement, cough (37/48, 77.1%) and fever (17/48, 35.4%) were the most common presenting symptoms in immunocompromised patients. More immunocompetent patients (15/38, 39.5%) were asymptomatic than immunocompromised patients (3/48, 6.3%) (P = 0.000). The most common presenting symptoms were headache (39/50, 78.0%) and fever (25/50, 50.0%) in the patients with CNS involvement. The most frequently underlying diseases or factors of immunocompromised patients were HIV infection (22/94, 23.4%), immune system disease (16/94, 17.0%), use of immunosuppressive drugs or corticosteroids (16/94, 17.0%) (see Table 2).

**Table 1:** Demographic and clinical characteristics in immunocompetent and immunocompromised pulmonary cryptococcosis patients.

Variables	Immunocompromised Patients (N = 94)(n,%)	Immunocompetent Patients (N = 42)(n,%)	P-value
**Sex**			
Male	64 (68.1)	23 (54.8)	0.176
Female	30 (31.9)	19 (45.2)	
**Age** (years)			
≤30	7 (7.5)	5 (11.9)	0.091
31–60	60 (63.8)	32 (76.2)	
å 60	27 (28.7)	5 (11.9)	
**CNS involvement**	46 (48.9)	4 (9.5)	**0.000**
**Exposure history (pigeon droppings)**	14 (14.9)	12 (28.6)	0.097
**Underlying diseases and factors**	88 (93.6)	0 (0.0)	**0.000**
**Presenting symptoms and signs (CNS non-involvement)**	N = 48	N = 38	
Asymptomatic	3 (6.3)	15 (39.5)	**0.000**
Cough	37 (77.1)	16 (42.1)	**0.002**
Fever	17 (35.4)	3 (7.9)	**0.004**
Chest pain	10 (20.8)	5 (13.2)	0.404
Shortness of breath	12 (25.0)	4 (10.5)	0.102
**Presenting symptoms and signs (CNS involvement)**			
	N = 46	N = 4	
Headache	36 (78.3)	3 (75.0)	NA
Fever	23 (50.0)	2 (50.0)	
Cough	9 (19.6)	0 (0.0)	
Vomiting	15 (32.6)	2 (50.0)	
Altered mental status	9 (19.6)	1 (25.0)	

CNS: central nervous system; NA: not applied.

**Table 2:** The underlying diseases and factors of immunocompromised pulmonary cryptococcosis patients.

Underlying diseases and factors	Patients (N = 94)(n,%)
HIV infection	22 (23.4)
Immune system disease	20 (21.3)
Immunosuppressive drugs or corticosteroids	16 (17.0)
Diabetes mellitus	10 (10.6)
Chronic lung disease	10 (10.6)
Hematologic disease	8 (8.5)
Chronic kidney disease	7 (7.4)
Organ transplantation	5 (5.3)
Solid tumor	5 (5.3)
Hepatobiliary diseases	6 (6.4)
Hepatitis B carriers	2(2.1)

### Radiological findings

**Figure 1:**
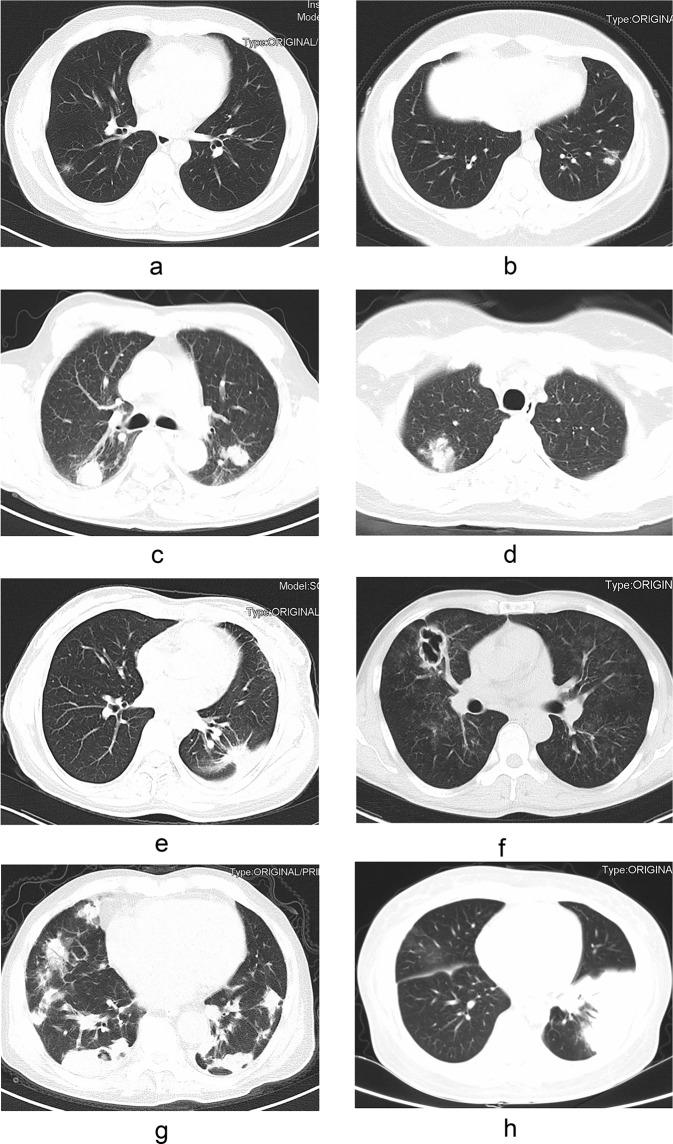
The findings of chest computed tomography of pulmonary crypotoccosis (immunocompetent patients: (**a**–**e**); immunocompromised patients: (**f**–**h**): (**a**) ground glass attenuation; (**b**) a single small nodule; (**c**) multiple lung nodules; (**d**) patchy shadows with air bronchogram; (**e**) nodular shadow with spiculate boundary; (**f**) pulmonary cavity; (**g**) scattered irregular patchy consolidation, nodules and mass shadows in bilateral lungs, and formation of cavities in a few lesions; (**h**) shadows of miliary nodules in bilateral lungs.

Chest CT was performed in all these patients. The imaging manifestations were diverse, as shown in Fig. 1. Table 3 shows the details of chest CT findings in immunocompromised and immunocompetent patients. The most common manifestations were pulmonary nodules (93/136, 68.4%) and patchy shadows (56/136, 41.2%). Multiple pulmonary nodules, ground-glass attenuation/interstitial changes and cavitation were more common in immunocompromised patients (P <0.05). The involvement rate of bilateral lungs was 57.4% (78/136), which mostly involving left lower lung. Lesions were more likely to involve bilateral lungs in immunocompromised patients (P <0.05). A total of 47 patients were misdiagnosed as tuberculosis or tumor. Immunocompromised PC patients were more likely to be misdiagnosed as tuberculosis than immunocompetent patients, and tumor was more likely to be considered in immunocompetent patients, but there was no significant difference (P >0.05).

**Table 3:** Chest CT findings in immunocompetent and immunocompromised pulmonary cryptococcosis patients.

Findings	Total (N = 136)(n,%)	Immunocompromised Patients (N = 94)(n,%)	Immunocompetent Patients (N = 42) (n,%)	P-value
**Lesion characterization**				
Pulmonary nodules	93 (68.4)	60 (63.8)	33 (78.6)	0.111
Solitary	16 (17.2)	4 (6.7)	12 (36.4)	**0.001**
Multiple	77 (82.8)	56 (93.3)	21 (63.6)	
Patchy shadows	56 (41.2)	40 (42.6)	16 (38.1)	0.707
Ground-glass attenuation /interstitial changes	18 (13.2)	17 (18.1)	1 (2.4)	**0.012**
Consolidations	28 (20.6)	19 (20.2)	9 (21.4)	1.000
Cavitation	35 (25.7)	31 (33.0)	4 (9.5)	**0.005**
Pleural effusion	20 (14.7)	14 (14.9)	6 (14.3)	1.000
Pleural thickening/adhesions	25 (18.4)	19 (20.2)	6 (14.3)	0.480
Enlarged lymph nodes	15 (11.0)	12 (12.8)	3 (7.1)	0.393
**Lesion area**				
Right lung	30 (23.5)	15 (16.0)	15 (35.7)	**0.014**
Left lung	26 (19.1)	16 (17.0)	10 (23.8)	0.355
Bilateral lungs	78 (57.4)	61 (64.9)	17 (40.5)	**0.009**
**Lesion area**				
Upper lung	89 (65.4)	67 (71.3)	22 (52.4)	0.050
Middle lung	58 (42.6)	44 (46.8)	14 (33.3)	0.189
Lower lung	115 (84.6)	80 (85.1)	35 (83.3)	0.801
**Lesion area**				
Right upper lobe	68 (50.0)	53 (56.4)	16 (38.1)	0.063
Right middle lobe	55 (40.4)	42 (44.7)	13 (31.0)	0.185
Right lower lobe	50 (36.8)	30 (31.9)	20 (47.6)	0.087
Left upper lobe	71 (52.2)	54 (57.4)	17 (40.5)	0.094
Left lower lobe	86 (63.2)	64 (68.1)	22 (52.4)	0.087
**Suspected tuberculosis**	17 (12.5)	15 (16.0)	2 (4.8)	0.069
**Suspected tumor**	30 (22.1)	17 (18.1)	13 (31.0)	0.096

### Diagnostic procedures for histopathologically confirmed patients

A total of 72 cases of PC were diagnosed by histopathology (as shown in Table 4). More immunocompetent patients were diagnosed by surgical biopsy, and percutaneous lung biopsy was more likely to be adopted in immunocompromised patients (P <0.05).

**Table 4:** Diagnostic procedures for histopathologically confirmed patients with pulmonary cryptococcosis.

Diagnostic method	Total (N = 72) (n,%)	Immunocompromised Patients (N = 38)(n,%)	Immunocompetent Patients (N = 34) (n,%)	P-value
Transbronchial lung biopsy	11 (15.3)	9 (23.7)	2 (5.9)	0.050
Percutaneous lung biopsy	31 (43.0)	23 (60.5)	8 (23.5)	0.002
Surgical lung biopsy	30 (41.7)	6 (15.8)	24 (70.6)	0.000

### Treatment strategies and clinical outcome

A total of 40.4% (38/94) and 35.7% (15/42) of immunocompromised and immunocompetent patients received treatment within 30 days after the onset of symptoms, respectively. There was no significant difference in the time from onset of symptoms to treatment/diagnosis between immunocompromised and immunocompetent patients (P > 0.05). Nine patients did not receive treatment after diagnosis. Treatment for other patients were categorized as surgery, antifungal drugs, or both. Most patients (104/136, 76.5%) were treated with antifungal drugs alone, especially immunocompromised patients. More immunocompetent patients (15/42, 35.7%) were treated with surgery and postsurgical medication than immunocompromised patients (5/94, 5.3%). Among CNS involved patients, most of them (41/50, 82.0%) were also given drug therapy alone. Surgery and drug therapy were combined in 39.5% (15/38) patients without CNS involvement. Among CNS uninvolved patients, more immunocompetent patients (30/37, 81.1%) were treated with fluconazole monotherapy than immunocompromised patients (29/45, 64.4%). The duration of the treatment ranged from 1 week to 2.5 years. The improvement rate in immunocompetent patients were higher than that of immunocompromised patients (P < 0.05), as shown in Table 5.

**Table 5:** Therapeutic regimen and treatment outcome of 136 patients with pulmonary cryptococcosis.

Variables	Immunocompromised Patients (N = 94)(n,%)	Immunocompetent Patients (N = 42)(n,%)	P-value	Improved outcome		P-value
Time from onset of symptoms to treatment/diagnosis (d)				Immunocompromised Patients (n,%)	Immunocompetent Patients (n,%)	
0–30	38 (40.4)	15 (35.7)	0.859	19 (19/38, 50.0)	15 (15/15, 100.0)	**0.000**
31–90	33(35.1)	15 (35.7)		20 (20/33, 60.6)	15 (15/15, 100.0)	**0.004**
91–180	14 (14.9)	6 (14.3)		7 (7/14, 50.0)	6 (6/6, 100.0)	0.051
>180	9 (9.6)	6 (14.3)		5 (5/9, 55.6)	6 (6/6, 100.0)	NA
**Time from onset of symptoms to treatment/diagnosis (d) (CNS non-involvement)**						
	**N** = **48**	**N** = **38**				
0–30	14 (29.2)	14 (36.8)	0.871	9 (9/14, 64.3)	14 (14/14, 100.0)	**0.041**
31–90	20 (41.7)	13 (34.2)		17 (17/20, 85.0)	13 (13/13, 100.0)	0.261
91–180	6 (15.0)	5 (13.2)		4 (4/6, 66.7)	5 (5/5, 100.0)	0.455
å 180	8 (16.6)	6 (15.8)		4 (4/8, 50.0)	6 (6/6, 100.0)	NA
**Time from onset of symptoms to treatment/diagnosis (d) (CNS involvement)**						
	**N** = **46**	**N** = **4**				
0–30	24 (52.2)	1 (25.0)	0.620	10 (10/24, 41.7)	1 (1/1, 100.0)	NA
31–90	12 (26.1)	2 (50.0)		3 (3/12, 25.0)	2 (2/2, 100.0)	
91–180	7 (15.2)	1 (25.0)		3 (3/7, 42.9)	1 (1/1, 100.0)	
å 180	3 (6.5)	0 (0.0)		1 (1/3, 33.3)	0	
**Treatment**						
No treatment	9 (9.6)	0 (0.0)	**0.000**	1 (1/9, 11.1)	0	NA
Surgery	2 (2.1)	1 (2.4)		1 (1/2, 50.0)	1 (1/1, 100.0)	NA
Antifungal drugs	78 (83.0)	26 (61.9)		50 (50/78, 64.1)	26 (26/26, 100.0)	**0.000**
Surgery + antifungal drugs	5 (5.3)	15 (35.7)		5 (5/5, 100.0)	15 (15/15, 100.0)	NA
**Treatment (CNS involvement)**						
	**N** = **46**	**N** = **4**				
No treatment	8 (17.4)	0 (0.0)	NA	0	0	NA
Surgery	0 (0.0)	0 (0.0)		0	0	
Antifungal drugs	37 (80.4)	4 (100.0)		22 (22/37, 59.5)	4 (4/4, 100.0)	
Surgery + antifungal drugs	1 (2.2)	0 (0.0)		1 (1/1, 100.0)	0	
**Treatment (CNS non-involvement)**						
	**N** = **48**	**N** = **38**				
No treatment	1 (2.1)	0 (0.0)	**0.006**	1 (1/1, 100.0)	0	NA
Surgery	2 (4.2)	1 (2.6)		1 (1/2, 50.0)	1 (1/1, 100.0)	NA
Antifungal drugs	41 (85.4)	22 (57.9)		28 (28/41, 68.3)	22 (22/22, 100.0)	**0.002**
Surgery + antifungal drugs	4 (8.3)	15 (39.5)		4 (4/4, 100.0)	15 (15/15, 100.0)	NA
**Antifungal treatment (CNS involvement)**						
	**N** = **38**	**N** = **4**				
FCZ	4 (10.5)	0 (0.0)	NA	0	0	NA
VCZ	0 (0.0)	0 (0.0)		0	0	
Itraconazole	0 (0.0)	0 (0.0)		0	0	
AmB + 5-FC	6 (15.8)	1 (25.0)		2 (2/6, 33.3)	1 (1/1, 100.0)	
AmB + FCZ/VCZ	8 (21.1)	0 (0.0)		5 (5/8, 62.5)	0	
FCZ/VCZ + 5-FC	4 (10.5)	1 (25.0)		2 (2/4, 50.0)	1 (1/1, 100.0)	
AmB + FCZ/VCZ + 5-FC	16 (42.1)	2 (50.0)		11 (11/16, 68.8)	2 (2/2, 100.0)	
**Antifungal treatment (CNS non-involvement)**						
	**N** = **45**	**N** = **37**				
FCZ	29 (64.4)	30 (81.1)	NA	24 (24/29, 82.8)	30 (30/30, 100.0)	**0.024**
VCZ	5 (11.1)	5 (13.5)		4 (4/5, 80.0)	5 (5/5, 100.0)	NA
Itraconazole	3 (6.7)	1 (2.7)		2 (2/3, 66.7)	1 (2/2, 100.0)	NA
AmB + 5-FC	0 (0.0)	0 (0.0)		0	0	NA
AmB + FCZ/VCZ	5 (11.1)	0 (0.0)		3 (3/5, 60.0)	0	NA
FCZ/VCZ + 5-FC	2 (4.4)	1 (2.7)		1 (1/2, 50.0)	1 (1/1, 100.0)	NA
AmB + FCZ/VCZ + 5-FC	1 (2.2)	0 (0.0)		1 (1/1, 100.0)	0	NA
**Outcome**						
Improved	53 (56.4)	42 (100.0)	**0.000**			
Failure	41 (43.6)	0 (0.0)				

AmB: amphotericin B; FCZ: fluconazole; VCZ: voriconazole; 5-FC: 5 fluorocytosine; NA:not applied.

### Radiologic outcomes after treatment

**Figure 2:**
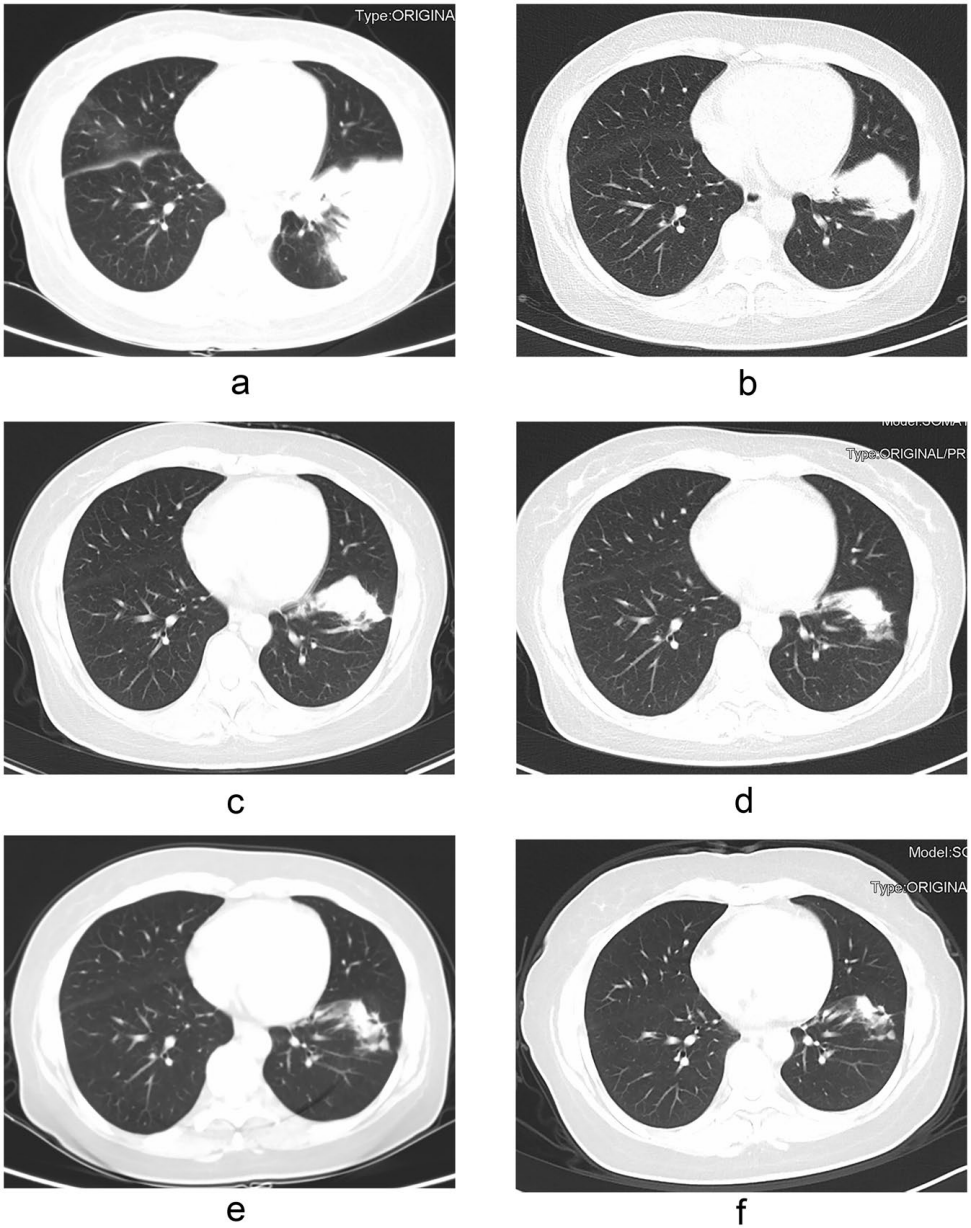
Pulmonary cryptococcosis in a 42-year-old woman with nephrotic syndrome and endometrial cancer after hysterectomy. CT imaging shows patchy consolidation in the left lower lobe of the lung (**a**). The lesion was significantly reduced after two months of treatment with fluconazole/voriconazole plus 5-fluorocytosine (**b**). The lesion continued to shrink after five months of treatment (**c**). The drug was discontinued after 13 months of treatment (**d**). The focus continued to shrink and there were still irregular soft tissue mass after 16 months (**e**) and 29 months (**f**) of drug withdrawal.

The available follow-up chest CT time in our hospital for immunocompromised patients was from 0.25 to 45 months, and 0.5 to 73 months for immunocompetent patients. After 3 months of treatment, 26 immunocompromised patients and 15 immunocompetent patients had follow-up chest CT scans in our hospital, 92.7% (38/41) of them have improved imaging findings. The time difference of CT imaging improvement in two groups was not statistically significant (P > 0.05) (see Table 6). The gradual radiologic improvement was found in the 43 patients who were treated with antifungal drugs even after their drug therapy was stopped. At the end of the follow-up period, complete radiologic remission could be found only in the six patients who underwent surgical resection. All the other patients had some residual abnormalities (such as high-density shadow, nodular shadow, pleural thickening, adhesion) and/or fibrotic scars. Figure 2 showed the changes of chest CT lesions during the follow-up of an immunocompromised patient with PC.

**Table 6:** Radiologic outcomes after 3 months of treatment in immunocompetent and immunocompromised pulmonary cryptococcosis patients.

Follow-up chest CT scans	Immunocompromised Patients (N = 29)	Immunocompetent Patients (N = 21)	P-value
Follow-up patients	26	15	NA
Improved patients	24	14	NA
The time of improvement (month)	0.25–3	0.5–3	0.623

NA: not applied.

## Discussion

Cryptococcosis is a potentially serious fungal infection and it mainly occurs in immunocompromised populations such as HIV positive patients^16^. This study also found that PC is more likely to occur in immunocompromised hosts. In this study, 69.1% (94/136) of the patients were immunocompromised. However, many previous studies have reported that cryptococcosis was more common in immunocompetent patients in China^17–19^. Another recent report showed 40.4% PC patients had normal immune function^12^. Although some patients with cryptococcosis seemed to be immunocompetent, some immune gene characteristics such as mannose-binding lectin (MBL) polymorphisms, FCGR (Fc-gamma receptors FccRs coding genes) 2B 232I/T genotype, FccRIIB polymorphisms, specific immunological status and/or a particular lifestyle were associated with these individuals^8–10,20^. Producing abundant anti-GM-CSF antibodies to perturb the function of phagocytes and macrophages maybe another impressionable factor for cryptococcosis in otherwise immunocompetent patients^21,22^. Animal experiments had shown that humoral immunodeficiency mice were susceptible to *C. neoformans*^23^. Therefore, how to correctly evaluate immune function of patients is a problem that needs to be solved. The high peak age of the patients with PC was from 31 to 60 years (92/136, 67.6%), regardless of their immune status. For immunocompetent patients, they may have more exposure to cryptococcal spores such as direct or indirect exposure to pigeon droppings and fungal contaminants at that age.

The common clinical manifestations of PC patients were cough, expectoration, fever, chest pain and shortness of breath. Headache, vomiting, altered mental status could occur in patients with meningitis. Immunocompromised patients without CNS involvement were more prone to have cough and fever, immunocompetent patients without CNS involvement were more likely to be asymptomatic, which is similar to previous research results^19,24,25^. HIV infections, immunosuppressant utilization and autoimmune diseases were common predisposing factors for immunocompromised patients. Many previous reports had similar results^17,26^. However, a review report showed tuberculosis and liver diseases were also the common underlying diseases in cryptococcosis in China^27^. Impaired cellular immune function is a common feature of these patients^28–30^. CD4+ T lymphocytes play an important role in the prevention of cryptococcus infection. Impaired T lymphocyte function may affect chemotaxis of granulocytes and macrophages and clearance of *Cryptococcus*^31^.

The most frequent imaging findings of PC were pulmonary nodules and patchy shadows. As a result, most patients were misdiagnosed as bacterial pneumonia and received antibiotic treatment before they were diagnosed with cryptococcosis. Host immune status is an important influencing factor for chest CT findings of PC. Extensive pulmonary involvement and cavitation were significantly more common in immunocompromised patients than immunocompetent patients in this study. Similar results were reported in many previous reports^7,32,33^. However more cases were included in our study. Except for bacterial pneumonia, our study also found that immunocompromised PC patients were more susceptible to be misdiagnosed as tuberculosis and immunocompetent PC patients might be misdiagnosed as tumor. A previous study showed that PC was often misdiagnosed as tumors and tuberculosis based on CT signs^34^. *Cryptococcus* spores (2–3 µm in diameter) or less encapsulated yeast cells are easily inhaled into the bronchioles and terminal bronchioles, where they were engulfed by macrophages and formed granulomas below the pleura. Inflammatory cell infiltration could lead to vasculitis and ultimately cause coagulative necrosis. Thus, cavities were observed on CT^34^. In short, the radiological presentations of PC are diverse and nonspecific, hence these patients were easily misdiagnosed.

Positive culture for *Cryptococcus* is the most accurate mean of confirming the diagnosis of cryptococcosis. However, due to the previous low vigilance against fungal infections, the rate of culture detection was low. Histopathological examination is a sensitive method for confirming cryptococcosis^28^. More than half of the patients in this study were diagnosed by histopathology. Some noninvasive diagnostic tests such as CrAg test have been gradually applied to clinical practice in recent years. Some patients in our group underwent CrAg test (data not shown), which had helped us to consider cryptococcosis diagnosis. Increasing number of detection methods have led to an increase in the detection rate of cryptococcosis. Perhaps this is also a cause of the increased incidence of cryptococcosis in recent years, even in the case of a reduction in HIV-associated cryptococcosis with extensive antiretroviral therapy (ART) treatment. Our study also showed more immunocompromised patients were diagnosed with percutaneous lung biopsy, while immunocompetent patients were more likely to be diagnosed by surgical lung biopsy. Most of the previous studies only showed the diagnostic methods and diagnostic procedures related to PC^7,35,36^, and few studies compared the differences in methods for obtaining tissue samples during pathological diagnosis of PC patients with different immune status. It is possible that immunocompromised patients have more extensive lung lesions and worse conditions, so percutaneous lung puncture with less invasion is selected. The imaging manifestations of some immunocompetent patients were single lesions. They were suspected to be lung cancer and were admitted to the department of thoracic surgery for surgical resection of the lesion. Therefore, more immunocompetent patients were confirmed by surgical lung biopsy. For patients with a single lung lesion, noninvasive diagnostic tests including CrAg test and tumor markers should be combined to consider the possible diagnosis of these patients, and initial invasive operation should be carefully selected.

Our research also showed the length of time from onset of symptoms to treatment/ diagnosis did not affect the treatment outcome. The length of the time often depends on the severity of patient’s symptoms. Some patients still could achieve better therapeutic efficacy even if their treatment was delayed. Most of these patients have mild or no symptoms, which generally developed slowly. Maybe the severity of the disease is a more important impact factor on the treatment outcome than the timely treatment. Most PC patients were treated with antifungal drugs alone. Nine patients did not receive antifungal therapy because by the time the diagnosis was made they were running out of time for treatment. Antifungal therapy is the main treatment for cryptococcosis^13,14,37^. Out of the overall 136 cases, 104 patients were treated with antifungal drugs alone and had good response. For PC patients without CNS involvement, our data suggested that fluconazole monotherapy has a good clinical effect. The most commonly used induction therapy is amphotericin B, 5-flucytosine with fluconazole or voriconazole in our patients with CNS involvement. This treatment combination has rarely been mentioned in previous studies, especially in HIV-negative patients^7,28^. On the other hand, the treatment duration of induction therapy is also a concern. Because, in our experience, induction therapy with amphotericin B plus 5-flucytosine and induction therapy lasting less than 4 weeks often lead to treatment failure, especially for immunocompromised patients, with continuous positive smear of *Cryptococcus* in CSF and continuous high titer test of CrAg. Maybe because of the methods we used to manage the elevated intracranial pressure were different from the ways the guidelines recommend^13,14^. Mannitol was used more often than percutaneous lumbar drainage or ventriculoperitoneal shunt to reduce intracranial pressure in our clinical practice because secondary infection and pipe blockage are common clinical problems after catheterization. Induction therapy with voriconazole plus 5-flucytosine also had a good therapeutic effect in the treatment of cryptococcal meningitis in this study. A previous study has shown that voriconazole was superior to other antifungal drugs in controlling cryptococcal infections^38^. Voriconazole has unique advantages in anti-*Cryptococcus* including impeding the formation of *Cryptococcus* virulence factor melanin, increasing the inhibition and scavenging effect of immune effector cells on *Cryptococcus*, rare drug resistance and maximum synergistic effect with flucytosine^39–43^. Therefore, for patients with cryptococcosis, voriconazole is an alternative, especially those with impaired renal function or those who cannot tolerate amphotericin B. Further researches are needed.

The available chest CT follow-up time varied widely according to patient compliance and illness. A total of 38 patients (38/41, 92.7%) showed improvement in chest imaging within three months, and one patient showed improvement in chest CT after one week of treatment. One problem here is whether antifungal drugs can be discontinued in patients with PC when the imaging is still partially abnormal after a period of treatment^44^. Our research showed that radiographic abnormalities would gradually improve even if antifungal drugs were discontinued. Until the end of follow-up, most nonsurgical patients still had some residual radiographic abnormalities, similar to those found in tuberculosis^45^, which may be related to the displacement or drainage of necrotic lung parenchyma and/or fungal gelatinous aggregation^46^. However, some previous studies have shown that complete remission was possible in most patients^7,45^. Other studies also showed that complete radiologic remission is difficult to achieve after drug therapy for PC patients^47,48^.

This study has some limitations. First, it was a retrospective study and only part of patients had complete follow-up data. Second, the detection rate of tissue culture after surgical resection was low, and the pathological diagnosis was mainly determined by morphology and special staining. It is important to raise the awareness of all doctors, especially surgeons, about tissue culture or molecular etiological diagnosis.

## Conclusions

This study showed that the most common chest CT manifestations in patients with PC were pulmonary nodules and patchy shadows. PC was easily misdiagnosed as tuberculosis in immunocompromised patients, while those immunocompetent patients were more likely to be misdiagnosed as tumor according to imaging findings. Initial invasive operation should be carefully selected in immunocompetent patients with a single lung lesion to reduce unnecessary lobectomy. Most PC patients without CNS involvement had good clinical outcomes with fluconazole monotherapy, especially the immunocompetent patients. The chest imaging abnormalities in PC patients might continue to improve after a period of treatment.

## Data Availability

All data generated or analysed during this study are included in this published article.
